# Biorheological Model on Flow of Herschel-Bulkley Fluid through a Tapered Arterial Stenosis with Dilatation

**DOI:** 10.1155/2015/406195

**Published:** 2015-03-05

**Authors:** S. Priyadharshini, R. Ponalagusamy

**Affiliations:** Department of Mathematics, National Institute of Technology, Tiruchirappalli, Tamilnadu 620015, India

## Abstract

An analysis of blood flow through a tapered artery with stenosis and dilatation has been carried out where the blood is treated as incompressible Herschel-Bulkley fluid. A comparison between numerical values and analytical values of pressure gradient at the midpoint of stenotic region shows that the analytical expression for pressure gradient works well for the values of yield stress till 2.4. The wall shear stress and flow resistance increase significantly with axial distance and the increase is more in the case of converging tapered artery. A comparison study of velocity profiles, wall shear stress, and flow resistance for Newtonian, power law, Bingham-plastic, and Herschel-Bulkley fluids shows that the variation is greater for Herschel-Bulkley fluid than the other fluids. The obtained velocity profiles have been compared with the experimental data and it is observed that blood behaves like a Herschel-Bulkley fluid rather than power law, Bingham, and Newtonian fluids. It is observed that, in the case of a tapered stenosed tube, the streamline pattern follows a convex pattern when we move from *r*/*R* = 0 to *r*/*R* = 1 and it follows a concave pattern when we move from *r*/*R* = 0 to *r*/*R* = −1. Further, it is of opposite behaviour in the case of a tapered dilatation tube which forms new information that is, for the first time, added to the literature.

## 1. Introduction

Blood flow through a stenosed artery is one of the important areas of research because a stenosed artery affects the entire cardiovascular system. Aortic stenosis causes chest pain and decreased blood flow to the brain resulting in loss of consciousness and heart failure which increases the risk of death. It is well known that fluid dynamical factors play a pivotal role in the formation and development of stenosis. Young [1] and Young and Tsai [2] studied the effects of stenosis on blood flow through arteries. Several investigators [3–10] analyzed the blood flow through a stenosed artery and have shown that the physical parameters affect the blood flow. Pulsatile flow of blood through a stenosed porous medium under the influence of periodic body acceleration considering blood as a Newtonian fluid has been studied by El-Shahed [11]. El-Shehawey et al. [12] have examined the pulsatile flow of blood through a tube considering blood as a Newtonian fluid taking into account the body acceleration and porosity of the tube. Sharma et al. [13] investigated the effects of radial variation of hematocrit and magnetic field on the flow of blood as a Newtonian fluid through a porous medium in a stenosed artery.

Viscoplastic materials are concentrated suspensions of solid particles or macromolecules and are classified as generalized Newtonian fluids. They flow like liquids when subjected to a stress above a critical value but respond as elastic or inelastic solids below this critical stress. According to the von Mises yield criterion, flow is assumed to occur when the second invariant of the stress exceeds the so-called yield stress [4]. It is understood that the important time-independent non-Newtonian fluid possessing a fluid behavior index (power law index) and yield values is the Herschel-Bulkley fluid, which has pivotal applications in polymer processing industries [12], developing blood oxygenators, and biomechanics [4]. Further, Herschel-Bulkley fluids include both shear thinning and shear thickening materials. The practical examples of such materials are greases, colloidal suspensions, starch pastes, tooth pastes, paints, and blood flow in an artery. These fluids have been useful as lubricant in roller bearing [13].

The non-Newtonian behavior of blood has been considered and studied by [14–17]. Chaturani and Samy [18] investigated the effects of non-Newtonian nature of blood treating it as a Casson's fluid and pulsatility on flow through a stenosed tube. The two-dimensional flow of power law fluid in stenosed arteries has been studied and the effect of power law index on the flow separation and reattachment point has been thoroughly investigated [19]. Nadeem et al. [20] and Ismail et al. [21] have investigated blood flow through a tapered artery with a stenosis assuming the blood as a non-Newtonian power law fluid model. They analyzed the influences of different parameters (power law index, flow rate, stenosis shape, and stenosis height) in different types of tapered arteries (converging tapered, diverging tapered, and nontaperted artery). Pincombe et al. [22] proposed a fully developed one-dimensional casson flow through a stenosed artery with multiple abnormal segments. They have studied the effects of multiple stenoses and poststenotic dilatation on non-Newtonian blood flow in small arteries. Scott Blair and Spanner [23] have suggested that blood obeys Casson's model only for moderate shear rate flows and that there is no difference between Casson's and Herschel-Bulkley plots over the range where Casson's plot is valid (for blood). Furthermore, Sacks et al. [24] have experimentally pointed out that blood shows the behavior characteristic of a combination of Bingham-plastic and pseudoplastic fluid-Herschel-Bulkley fluid with the fluid behavior index greater than unity. In view of the experimental observation [24] and suggestion made in [23], it is pertinent to consider the behavior of blood as a Herschel-Bulkley fluid.

The non-Newtonian aspects of blood flow through stenosed arteries have been studied by [25] treating blood as a Herschel-Bulkley fluid. Biswas and Laskar [26] have investigated the steady flow of blood as a Herschel-Bulkley fluid through a stenosed artery. In these studies, the combined effects of the rheology of blood as Herschel-Bulkley fluid model, stenosis height, dilatation depth, and tapering on the flow of blood have not been investigated. Hence, the aim of the present paper is to analyze the flow of Herschel-Bulkley fluid in a tapered artery with stenosis and dilatation (Figure 2). The expressions for velocity, wall shear stress, and flow resistance have been derived. The effects of parameters such as power law index, shear dependent nonlinear viscosity, stenotic height, taper angle, dilatation depth, and the yield stress on physiologically important quantities, namely, wall shear stress and flow resistance, are presented graphically.

## 2. Formulation of the Problem

**Figure 1:**
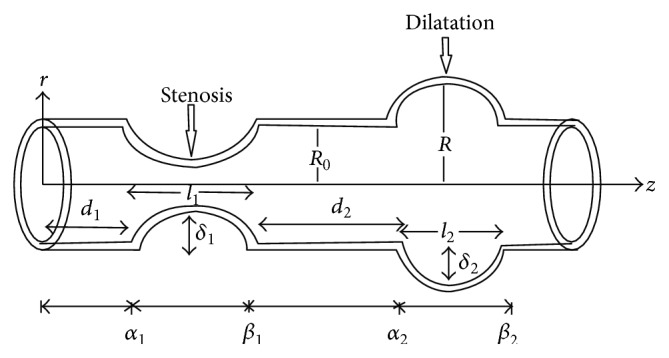
Geometry of an axially nonsymmetrical artery with stenosis and dilatation.

**Figure 2:**
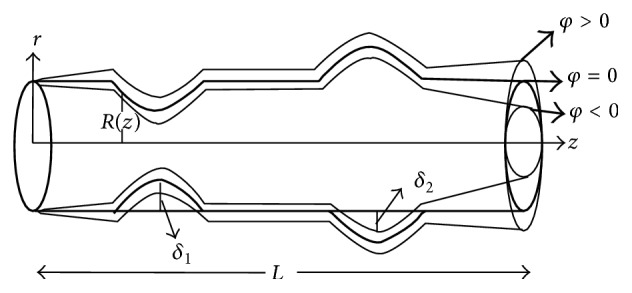
Geometry of the tapered artery with stenosis and dilatation for different taper angle.

Consider the steady and axially symmetric flow of an incompressible Herschel-Bulkley fluid lying in a tube having length *L* (Figures 1 and 2). We take the cylindrical coordinate system $\left(\bar{r},\bar{\theta },\bar{z}\right)$ in such a way that $\bar{u}$, $\bar{v}$, and $\bar{w}$ are the velocity components in $\bar{r}$, $\bar{\theta }$, and $\bar{z}$ directions, respectively. The equations governing the two-dimensional steady incompressible Herschel-Bulkley fluid are

(1) $$\begin{array}{c} \rho \left(\bar{u}\frac{\partial }{\partial \bar{r}}+\bar{w}\frac{\partial }{\partial \bar{z}}\right)\bar{u}=-\frac{\partial \bar{p}}{\partial \bar{r}}+\frac{1}{\bar{r}}\frac{\partial }{\partial \bar{r}}\left(\bar{r}{\bar{\tau }}_{\bar{r}\bar{r}}\right)+\frac{\partial {\bar{\tau }}_{\bar{r}\bar{z}}}{\partial \bar{z}}-\frac{{\bar{\tau }}_{\bar{\theta }\bar{\theta }}}{\bar{r}},\\ \rho \left(\bar{u}\frac{\partial }{\partial \bar{r}}+\bar{w}\frac{\partial }{\partial \bar{z}}\right)\bar{w}=-\frac{\partial \bar{p}}{\partial \bar{z}}+\frac{1}{\bar{r}}\frac{\partial }{\partial \bar{r}}\left(\bar{r}{\bar{\tau }}_{\bar{r}\bar{z}}\right)+\frac{\partial {\bar{\tau }}_{\bar{z}\bar{z}}}{\partial \bar{z}}. \end{array}$$

In the above equations the extra stress tensor $\bar{\tau }$ for Herschel-Bulkley fluid is defined as

(2) $$\begin{array}{c} \bar{\tau }=\bar{k}{\left(\bar{\dot{\gamma }}\right)}^{n-1}{\bar{\dot{\gamma }}}_{ij}+{\bar{\tau }}_{y}, \end{array}$$

where $\bar{k}$ is the consistency index, *n* is the power law index (or fluid behaviour index), ${\bar{\tau }}_{y}$ is the yield stress, and

(3) $$\begin{array}{c} \bar{\dot{\gamma }}=\sqrt{\frac{1}{2}\underset{i}{\sum }\underset{j}{\sum }{\bar{\dot{\gamma }}}_{ij}{\bar{\dot{\gamma }}}_{ij}}=\sqrt{\frac{1}{2}\pi }, \end{array}$$

where ${\bar{\gamma }}_{ij}$, *i*, *j* = 1,2, 3, is the rate of strain tensor component.

We introduce the nondimensional variables

(4) r=r¯R¯0,  z=z¯l¯,  w=w¯u¯0,  u=l¯u¯u¯0δ¯,p=R¯02p¯u¯0l¯μ¯,  Re=ρ¯R¯02u¯0μ¯,  τrr=l¯τ¯rru¯0μ¯,τrz=R¯0τ¯rzu¯0μ¯,  τzz=l¯τ¯zzu¯0μ¯,  τθθ=l¯τ¯θθu¯0μ¯,τy=τ¯yR¯0u¯0μ¯,  R(z)=R¯z¯R¯0,

where ${\bar{u}}_{0}$ is the average velocity of flow of Newtonian fluid, $\bar{l}=\min({\bar{l}}_{1},{\bar{l}}_{2})$, $\bar{\delta }=\max({\bar{\delta }}_{1},{\bar{\delta }}_{2})$, ${\bar{R}}_{0}$ is the radius of the normal artery, Re is the Reynolds number, $\bar{R}(\bar{z})$ is the radius of the abnormal artery, and $\bar{\mu }$ is the viscosity of Newtonian fluid. By assuming

(5) (i)  Reδ¯n1/n−1l¯≪1,(ii)  R¯0n1/n−1l¯~O(1),

the cases of mild stenosis $\left({\bar{\delta }}_{1}/{\bar{R}}_{0}\ll 1\right)$ and mild dilatation $\left({\bar{\delta }}_{2}/{\bar{R}}_{0}\ll 1\right)$, (1) with the help of (2) and (4) take the form

(6) $$\begin{array}{c} -\frac{\partial p}{\partial r}=0,\\ \frac{\partial p}{\partial z}=\frac{1}{r}\frac{\partial }{\partial r}\left(r\tau \right), \end{array}$$

where |*τ*| = *τ*_*y*_ + *k*(−∂*w*/∂*r*)^*n*^. The corresponding boundary conditions are

(7) i  τ  is  finite at  r=0ii  w=0 at  r=R.

The equations describing the geometry of the wall are

(8) R(z)=(1−ζz)1−δi2R0hhhhhhlh·1+cos⁡2πliz−αi−li2, iiiiiiiiiiiiiiiiiiiiiiiiiiiiiiiiiiiiiiiiiαi≤z≤βi1−ζz, iiiiiiiiiiiiiiiiiiiiiiiiiiiiiiiiiiiiiiiiiiiotherwise,

where *δ*_*i*_ is the maximum distance the *i*th abnormal segment projects into the lumen and is negative for aneurysms and positive for stenosis, *R* is the radius of the artery, and *ζ* = tan*ϕ*, where *ϕ* is the taper angle. For converging tapering *ϕ* becomes greater than 0, *ϕ* < 0 indicates the diverging tapering, and *ϕ* = 0 for the case of nontapered artery, *l*_*i*_ is the length of the *i*th abnormal segment, *α*_*i*_ denotes the distance from the origin to the commencement of the *i*th abnormal segment and is given by

(9) $$\begin{array}{c} \alpha_{i}=\left(\overset{i}{\underset{j=1}{\sum }}\left(d_{j}+l_{j}\right)\right)-l_{i}, \end{array}$$

*β*_*i*_ indicates the distance between the origin of the flow region and the end of the *i*th abnormal segment and is given by

(10) $$\begin{array}{c} \beta_{i}=\overset{i}{\underset{j=1}{\sum }}\left(d_{j}+l_{i}\right), \end{array}$$

and *d*_*i*_ is the distance separating the start of the *i*th abnormal segment from the end of the (*i* − 1)th or from the start of the segment if *i* = 1 [22].

## 3. Solution of the Problem

The exact solution for velocity field satisfying the boundary conditions can be written as

(11) w=nn+1Rn+1q(z)2k1/n ·1−RpRn+1/n−rR−RpRn+1/n,

where *q*(*z*) = −*dp*/*dz*.

The plug core velocity is given by

(12) $$\begin{array}{c} w_{p}=\left(\frac{n}{n+1}\right){\left(\frac{R^{n+1}q(z)}{2k}\right)}^{1/n}\left[{\left\{1-\frac{R_{p}}{R}\right\}}^{\left(n+1\right)/n}\right], \end{array}$$

where *R*_*p*_ is the radius of the plug core region and *R*_*p*_ = 2*τ*_*y*_/*q*(*z*).

Multiplying (11) by *r* and integrating with respect to *r*, the stream function *ψ*  (*w* = (1/*r*)(∂*ψ*/∂*r*), *u* = (−1/*r*)(∂*ψ*/∂*z*)) is obtained as

(13) ψ=nn+1Rn+1q(z)2k1/n ·1−RpRn+1/nr22−nrR2n+1rR−RpR2n+1/nhhhh+R2n22n+13n+1rR−RpR3n+1/n.

The volumetric flow rate is defined as

(14) $$\begin{array}{c} Q=2\overset{R}{\underset{0}{\int }}ru\left(r\right)dr. \end{array}$$

The total flow rate *Q* is defined as

(15) $$\begin{array}{c} Q=2\overset{R_{p}}{\underset{0}{\int }}ru_{p}dr+2\overset{R}{\underset{R_{p}}{\int }}ru\left(r\right)dr. \end{array}$$

Using (11), (12), and (15), we get

(16) Q=nR23n+1Rn+1q(z)2k1/n1−RpRn+1/n ·1+4n2n+1Rp2R+8n22n+1n+1Rp24R2.

The shear stress *τ* at the wall of the tapered arterial stenosis with dilatation (wall shear stress *τ*_*w*_) is defined as

(17) $$\begin{array}{c} \tau_{w}=\frac{R}{2}q\left(z\right). \end{array}$$

The flow resistance *λ* is defined as

(18) $$\begin{array}{c} \lambda =\overset{z}{\underset{0}{\int }}\frac{q\left(z\right)}{Q}dz, \end{array}$$

where *z* is any point of cross section of nonuniform tube along the axial direction.

**Case 1 .**

For any value of yield stress *τ*_*y*_, (16) can be rewritten as

(19) 3n+1nR2Qx3 =Rx−2τyn+12k1/n  ·x2+4n2n+1τyxR+8n22n+1n+1τy2R2,

where *x* = −*dp*/*dz*. For *Q* = 1.0, one can numerically compute the value of *x* (pressure gradient) from (19) for different values of the parameters. Equation (19) has been numerically solved for *x* using Newton-Raphson method.

**Case 2 .**

For small value of yield stress *τ*_*y*_/*τ*_*w*_ ≪ 1, the expression for pressure gradient can be obtained as

(20) −dpdz=21/nk1/n(3n+1)QnR3n+1/nn+2(3n+1)2n+1τyR +42n3+56n2+26n+42n+12n+1τy2nnR3n−12k3n+1nQn.

Using (17) and (20), the wall shear stress is obtained as

(21) τw=k1/n(3n+1)QnR3n+3n+12n+1τy +42n3+56n2+26n+42n+12(n+1)τy2nnR3n4k3n+1nQn.

Substituting (20) into (18), the analytical expression for flow resistance is obtained as

(22) $$\begin{array}{c} \lambda =a\overset{L}{\underset{0}{\int }}\frac{1}{R^{3n+1}}dz+b\overset{L}{\underset{0}{\int }}\frac{1}{R}dz+c\overset{L}{\underset{0}{\int }}R^{3n-1}dz, \end{array}$$

where

(23) a=1Qn−121/nk1/n(3n+1)nn,b=2(3n+1)τy(2n+1)Q,c=242n3+56n2+26n+42n+12n+1τy2nn2k3n+1nQn+1.

Considering the number of abnormal segments within an arterial segment as shown in Figure 1, we define *α*_*i*_ as the starting point and *β*_*i*_ as the ending point of each portion. Taking this into account (22) can be rewritten as

(24) λ=a∫0α1dz+∑i=1m∫αiβiR−(3n+1)dz+∑i=1m−1∫βiαi+1dz+∫βmLdz +b∫0α1dz+∑i=1m∫αiβiR−1dz+∑i=1m−1∫βiαi+1dz+∫βmLdz +c∫0α1dz+∑i=1m∫αiβiR3n−1dz+∑i=1m−1∫βiαi+1dz+∫βmLdz.

## 4. Discussion

A comparison between numerical values and analytical values of pressure gradient at the midpoint of stenotic region shows that, up to *τ*_*y*_ = 2.4, the maximum error is less than 1.4% and for dilatation region the maximum error is less than 6%. This is illustrated in Tables 1 and 2. This implies that the analytical expression for pressure gradient works well for the values of yield stress till 2.4.

**Figure 3:**
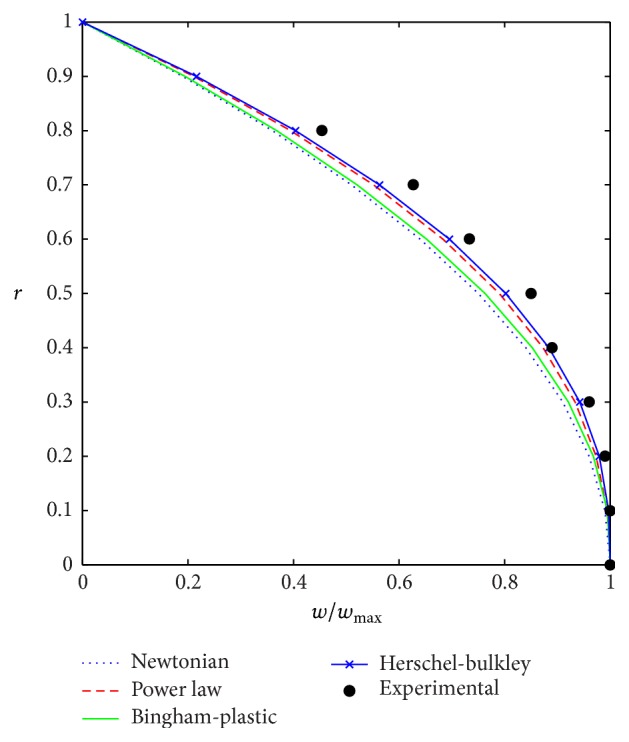
Comparison of velocity profiles for various fluids with experimental results.

A comparative study of velocity profiles for fluids such as Newtonian, power law, Bingham-plastic, and Herschel-Bulkley fluids is represented graphically in Figure 3. From Figure 3, it is observed that the velocity of Herschel-Bulkley fluid agrees with experimental values compared to that of the other fluids.

**Figure 4:**
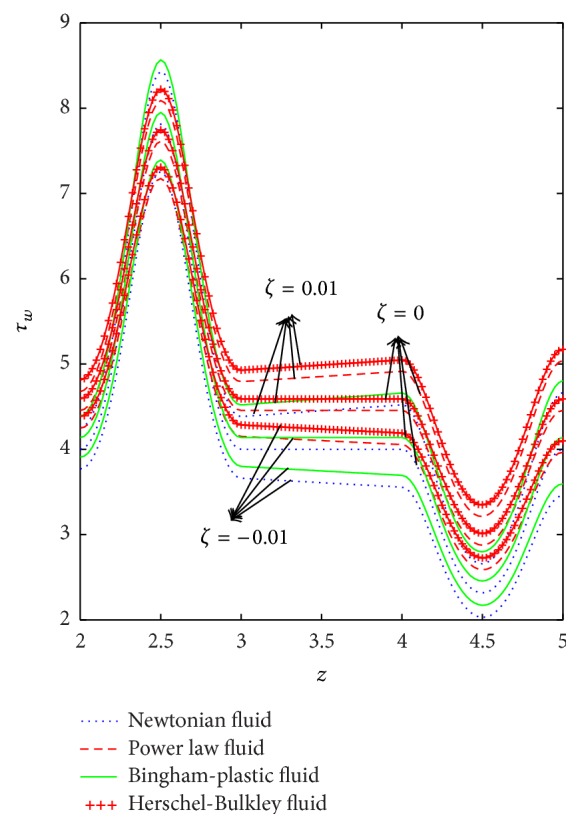
Axial variation of wall shear stress for Newtonian, power law, Bingham-plastic, and Herschel-Bulkley fluids with different values of *ζ*.

**Figure 5 fig5:**
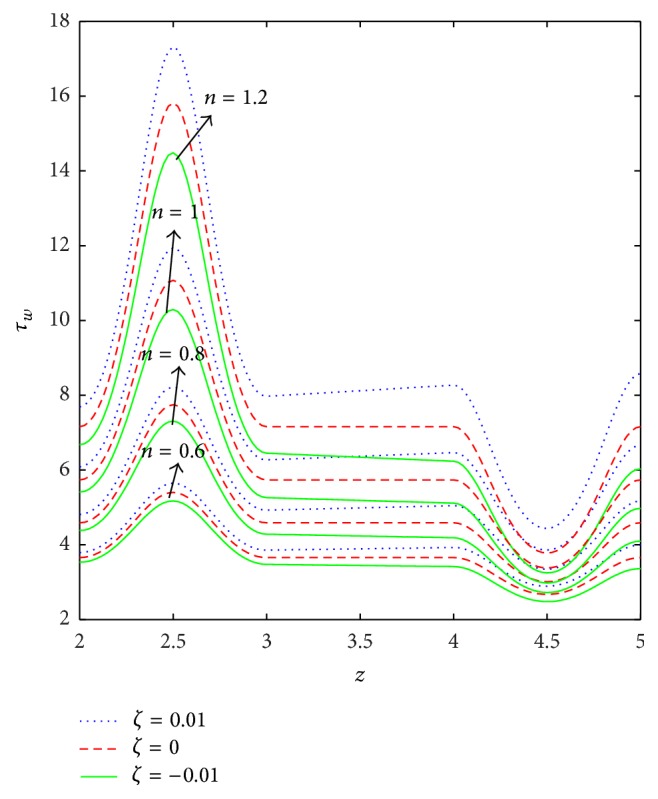
Axial variation of wall shear stress (*τ*_*w*_) for different values of power law index (*n*) taking *k* = 1.4, *δ* = 0.2, and *τ*_*y*_ = 0.1.

**Figure 6 fig6:**
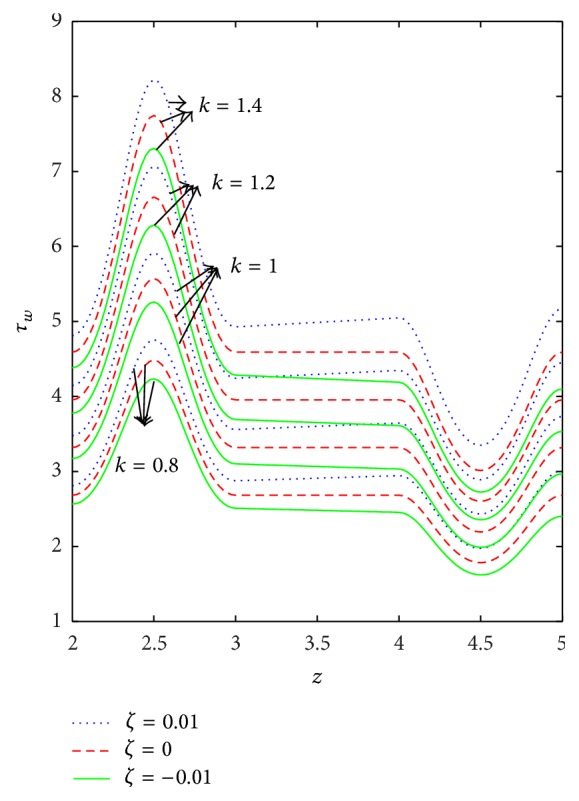
Axial variation of wall shear stress (*τ*_*w*_) for different values of consistency index (*k*) taking *n* = 0.8 and *τ*_*y*_ = 0.1.

**Figure 7 fig7:**
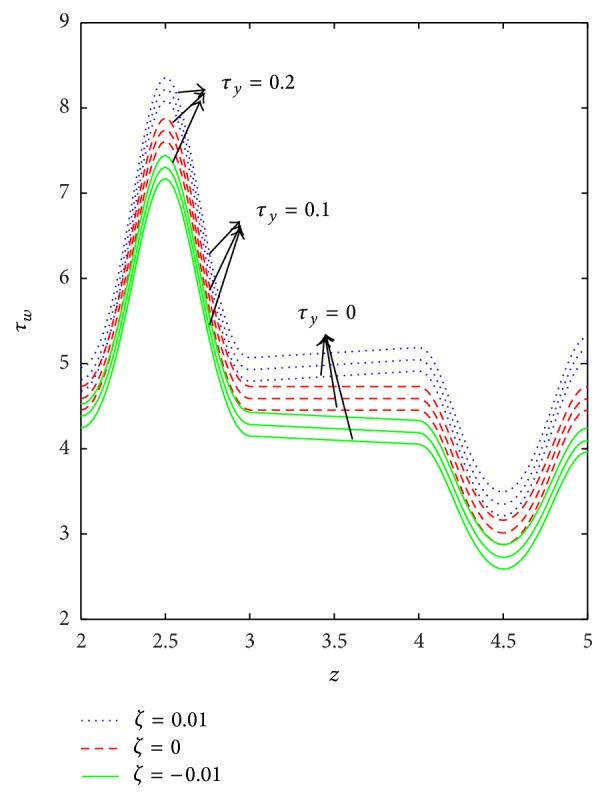
Variation of wall shear stress (*τ*_*w*_) with respect to axial distance for different values of *τ*_*y*_ taking *n* = 0.8, *k* = 1.4, and *δ* = 0.2.

**Figure 8:**
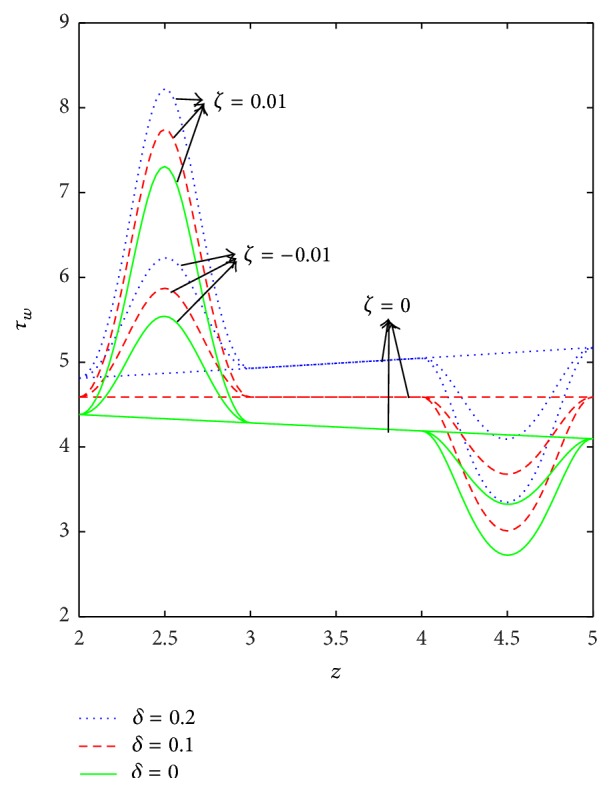
Axial variation of wall shear stress (*τ*_*w*_) for different values of *δ* by taking *n* = 0.8, *k* = 1.4, and *τ*_*y*_ = 0.1.

The variation of wall shear stress (WSS) with respect to axial distance for the case of a converging tapered, not tapered, and diverging tapered arterial stenosis with dilatation is displayed in Figures 4 [Fig fig5] [Fig fig6] [Fig fig7]–8. WSSs of fluids such as Newtonian, power law, Bingham-plastic, and Herschel-Bulkley fluids are compared in Figure 4. It is important to note that WSS increases in the upstream of the stenotic region (*z* = 2 to 2.5), reaches maximum at the midpoint (*z* = 2.5), and decreases in the downstream of region (*z* = 2.5 to 3), while, in the dilatation region, WSS decreases as *z* varies from 4 to 4.5, reaches minimum at the midpoint (*z* = 4.5), and increases in the region (*z* = 4.5 to 5). In the case of stenosis, increase is more for converging tapered artery (*ζ* = 0.01) as compared to the case of not tapered (*ζ* = 0) and diverging tapered (*ζ* = −0.01) artery. It is observed from the view of variation of WSS around the midpoint of stenotic region that the effect of the presence of stenosis is higher on the rheology of blood as Bingham fluid model in comparison with the rheology of blood as Newtonian, Hershel-Bulkley, and power law fluid models, respectively. It is important to observe from Figure 5 that the power law index (*n*) plays a significant role in stenotic region (*z* = 2 to 3) since the percentage of variation in WSS is higher for stenosis as compared to the case of dilatation.

Axial variation of WSS with respect to yield stress in the case of converging tapered, not tapered, and diverging tapered arterial stenosis with dilatation is displayed in Figure 7. Increase in yield stress causes wall shear stress to increase and the variation is more in the stenotic region than in the dilatation region. The effect of stenotic height on WSS has been investigated in Figure 8. As stenotic height increases, WSS increases in the stenotic region while it decreases in the dilatation region. When there is no stenosis, WSS increases linearly with respect to the axial distance. It is observed that the stenotic height plays a predominant role in increasing the WSS. The variation is more in the case of converging tapered than not tapered and diverging tapered arteries.

**Figure 9:**
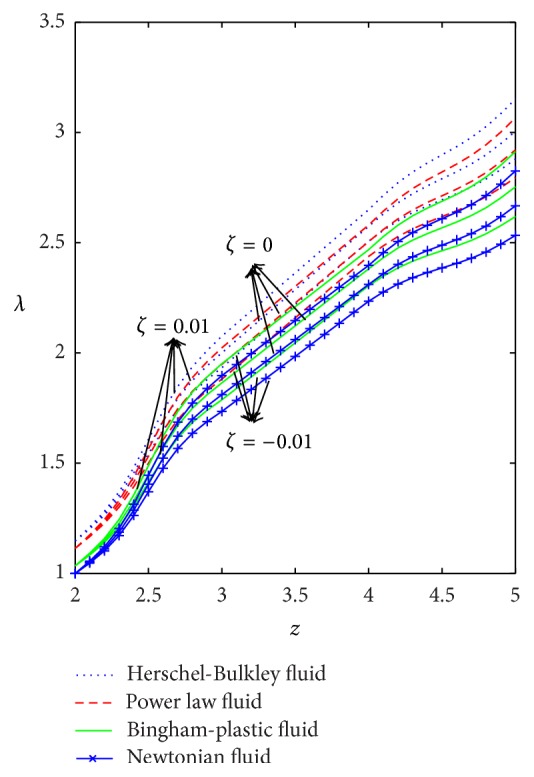
Variation of flow resistance (*λ*) for various fluids with respect to axial distance taking *δ* = 0.2.

**Figure 10:**
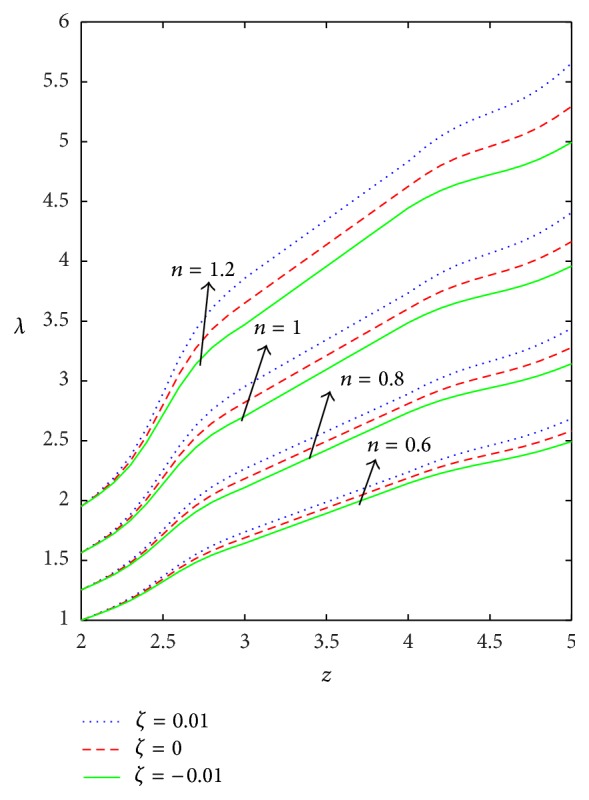
Axial variation of flow resistance (*λ*) for different values of *n* taking *k* = 1.2, *τ*_*y*_ = 0.1, and *δ* = 0.2.

**Figure 11:**
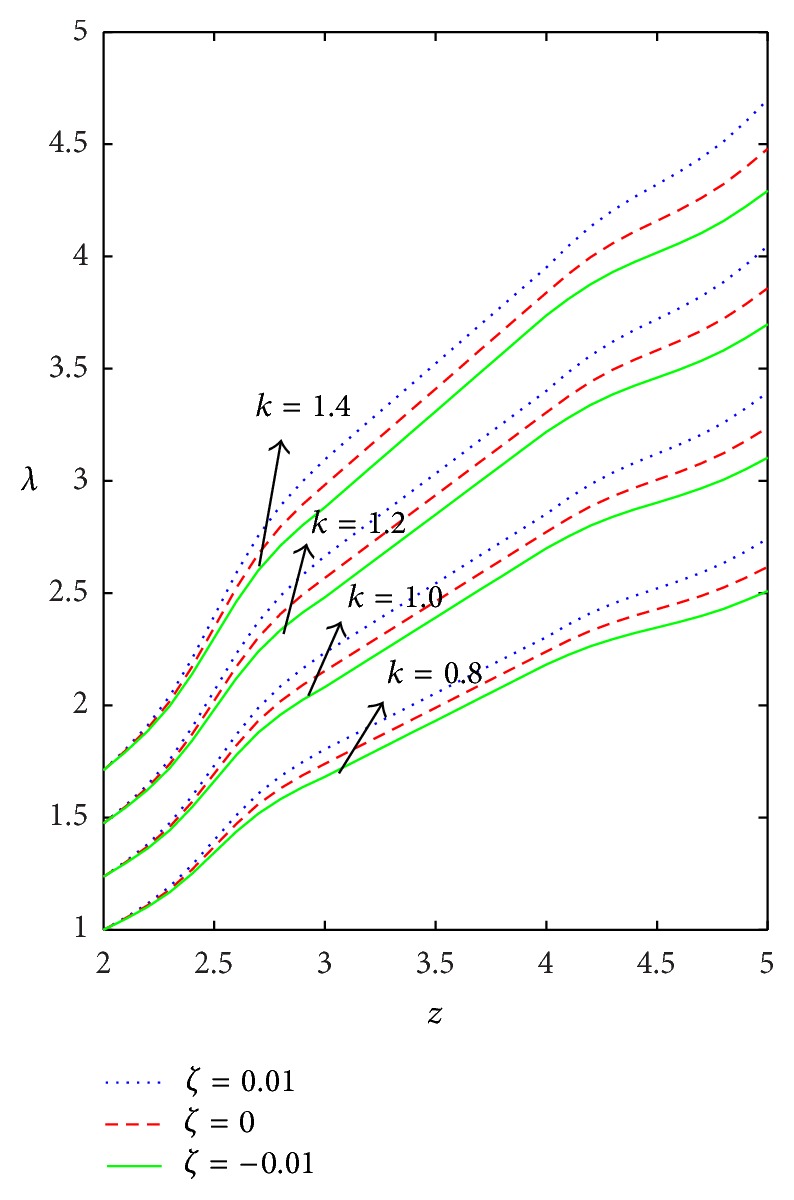
Axial variation of flow resistance (*λ*) for different values of *k* taking *n* = 0.8, *τ*_*y*_ = 0.1, and *δ* = 0.2.

**Figure 12:**
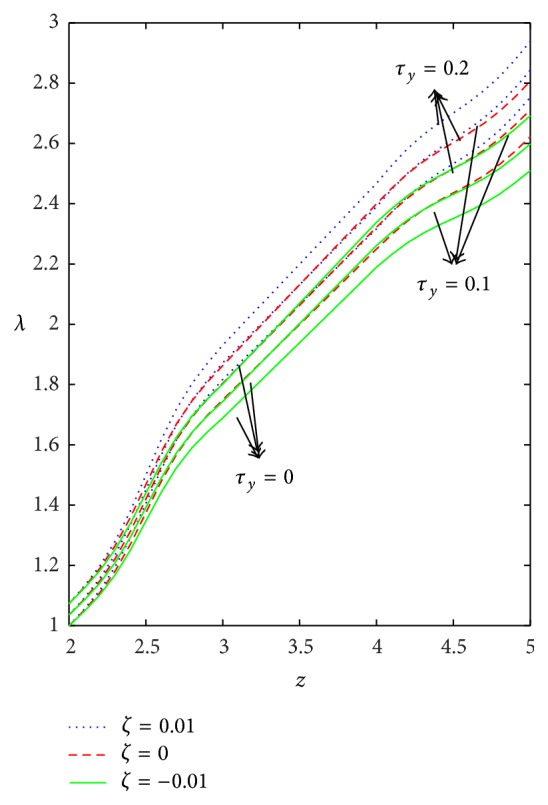
Axial variation of flow resistance (*λ*) for different values of yield stress (*τ*_*y*_) taking *n* = 0.8, *k* = 1.2, and *δ* = 0.2.

**Figure 13:**
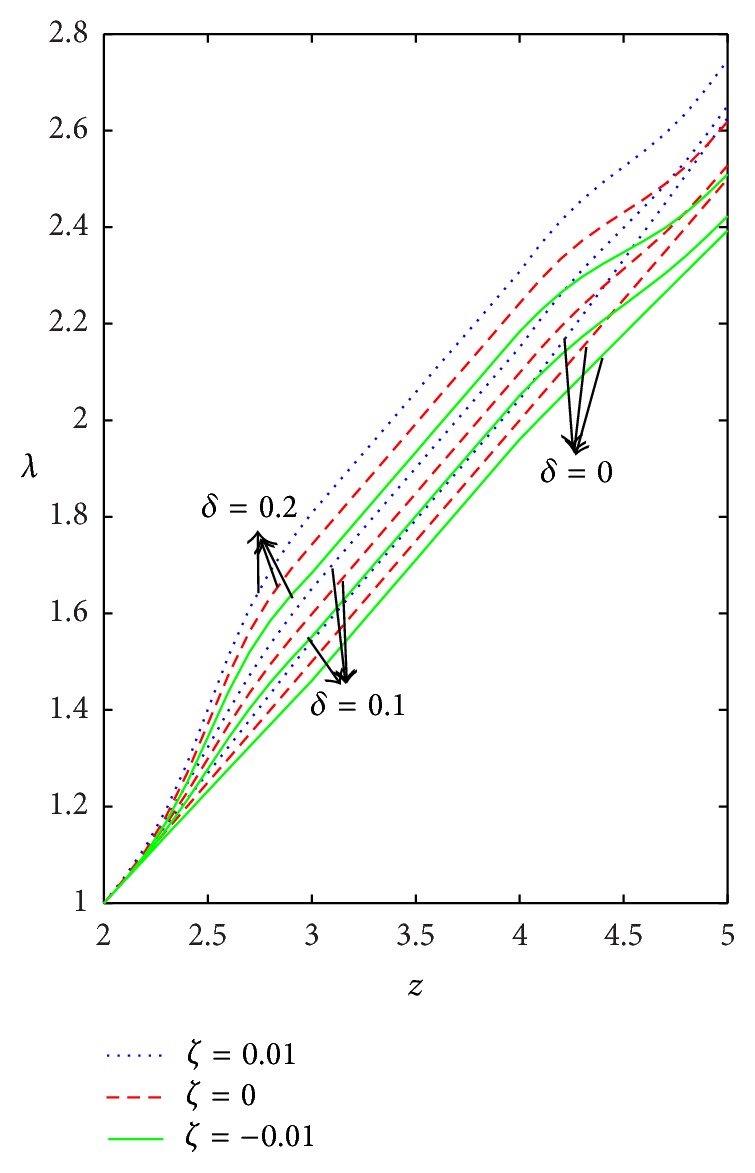
Variation of flow resistance (*λ*) with respect to axial distance for different values of *δ* taking *n* = 0.8, *k* = 1.2, and *τ*_*y*_ = 0.1.

Figures 9–13 are prepared to see the variation of resistance to flow with respect to the axial distance in the case of converging tapered, not tapered, and diverging tapered arterial stenosis with dilatation. A comparative study of flow resistance for Newtonian, power law, Bingham-plastic, and Herschel-Bulkley fluids is depicted in Figure 9. Flow resistance increases significantly in the stenotic region (*z* = 2 to 3): the increase is more for Herschel-Bulkley fluid and comparatively less for Newtonian fluid. Flow resistance decreases with the axial distance in the dilatation region. The variation of flow resistance for power law and Bingham-plastic is lesser when compared with Herschel-Bulkley and greater when compared with Newtonian fluid. Figures 10 and 11 depict that flow resistance increases as *n* and *k* increases. Increase in power law index causes flow resistance to increase significantly as compared to the consistency index (*k*). The effect of yield stress on flow resistance having other parameters fixed has been studied from Figure 12. Flow resistance increases as yield stress increases and the variation caused by yield stress is less compared to other parameters.  Figure 13 shows that the flow resistance increases with stenotic height and its increase is more in the case of converging tapered artery.

**Figure 14:**
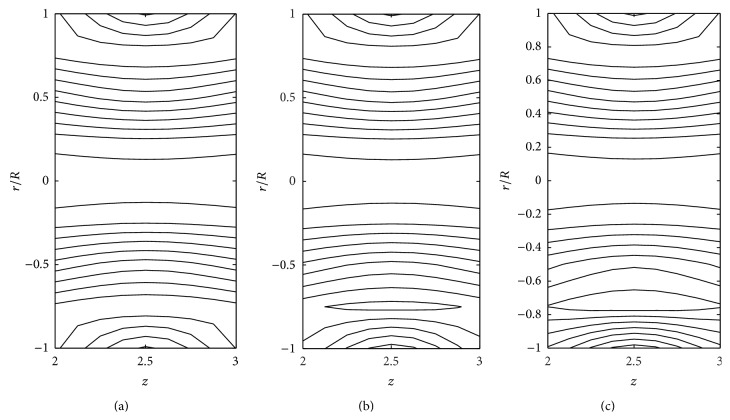
Stream lines for (a) Newtonian fluid, (b) Bingham-plastic fluid (*τ*_*y*_ = 0.1), and (c) Bingham-plastic fluid (*τ*_*y*_ = 0.4) taking *δ*_1_ = 0.2 and *ζ* = 0.01 in the stenotic region.

**Figure 15 fig15:**
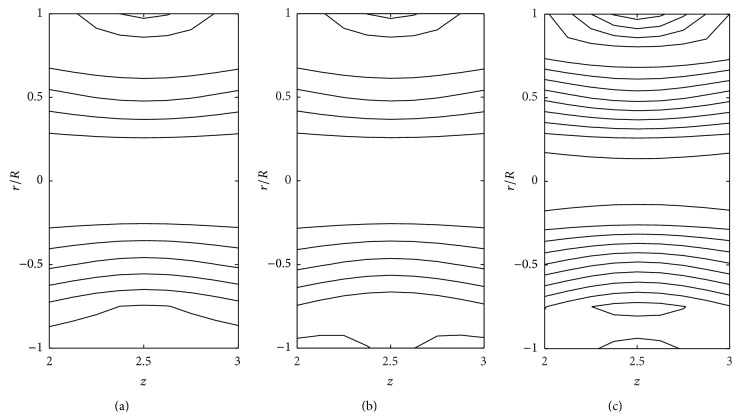
Stream lines for (a) power law fluid, (b) Herschel-Bulkley fluid (*τ*_*y*_ = 0.1), and (c) Herschel-Bulkley fluid (*τ*_*y*_ = 0.4) taking *δ*_1_ = 0.2 and *ζ* = 0.01 in the stenotic region.

**Figure 16 fig16:**
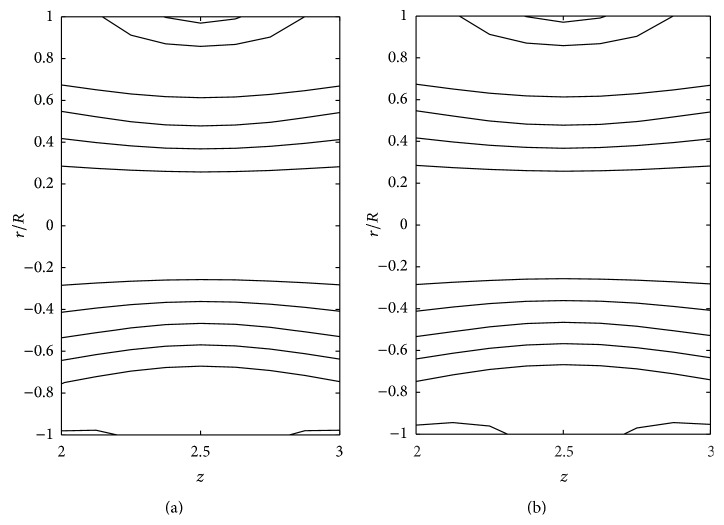
Stream lines for different values of *k*: (a) *k* = 1 and (b) *k* = 1.2 taking *δ*_1_ = 0.2, *ζ* = 0.01, *n* = 0.8, *k* = 1.4, and *τ*_*y*_ = 0.1 in the stenotic region.

**Figure 17 fig17:**
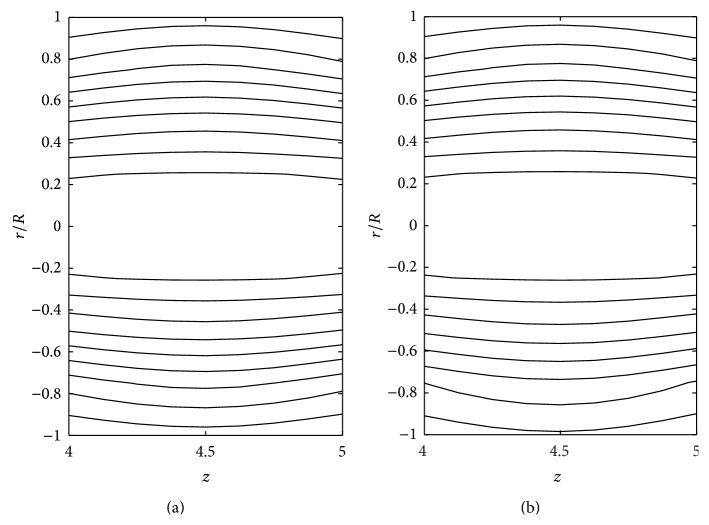
Stream lines for (a) Newtonian fluid and (b) Bingham-plastic fluid taking *δ*_2_ = −0.2 and *ζ* = 0.01 in the dilatation region.

**Figure 18 fig18:**
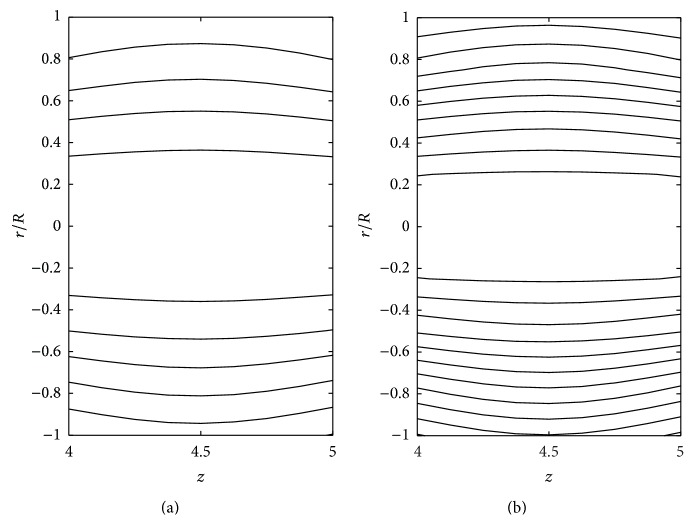
Stream lines for (a) power law fluid and (b) Herschel-Bulkley fluid taking *δ*_2_ = −0.2 and *ζ* = 0.01 in the dilatation region.

**Figure 19:**
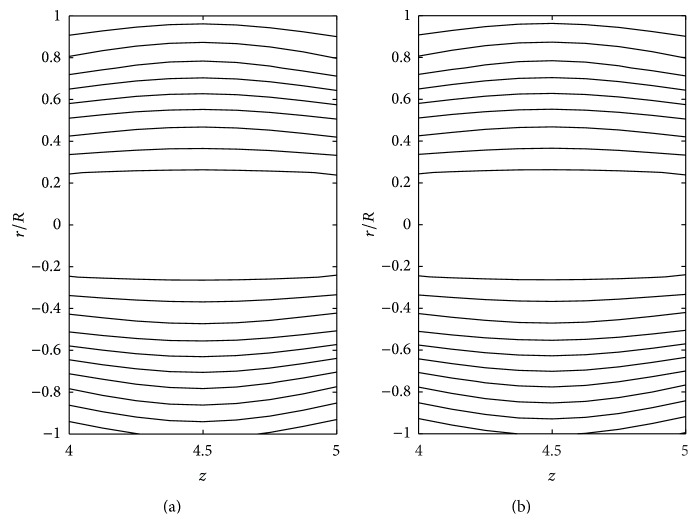
Stream lines for different values of *k*: (a) *k* = 1 and (b) *k* = 1.2 taking *n* = 0.8, *τ*_*y*_ = 0.1, *δ*_2_ = −0.2, and *ζ* = 0.01 in the dilatation region.

The effects of consistency index (*k*), power law index (*n*), and yield stress (*τ*_*y*_) on the stream line pattern have been examined and illustrated in Figures 14 [Fig fig15] [Fig fig16] [Fig fig17] [Fig fig18]–19. In the case of tapered stenosed tube (Figures 14–16), the non-Newtonian behaviour of blood plays a predominant role in the formation of trapping bolus. Increase in *k* or *n* does not cause a significant change in the stream line pattern. Increase in yield stress leads to a significant increase in the size of trapping bolus. It is observed that the parameters *k* and *n* are weak parameters in the sense that these parameters bring a small change in the stream line pattern in comparison with the yield stress. Figures 14–19 reveal that, in the case of a tapered stenosed tube, the stream line pattern follows a convex pattern when we move from *r*/*R* = 0 to *r*/*R* = 1 and it follows a concave pattern when we move from *r*/*R* = 0 to *r*/*R* = −1. Further, it is of opposite behaviour in the case of a tapered dilatation tube. In the case of dilatation, the variation in the stream line pattern corresponding to change in parameters is less due to lower pressure gradient. This has been illustrated in Figures 17–19.

## 5. Conclusion

This work presents a model of flow of an incompressible Herschel-Bulkley fluid through a tapered artery with stenosis and dilatation. In this paper, we conclude the following.

(i) Expressions for velocity profile, wall shear stress, and flow resistance are derived.
(ii) A comparison between numerical values and analytical values of pressure gradient at the midpoint of stenotic region shows that, up to *τ*_*y*_ = 2.4, the maximum error is less than 1.4% and, for dilatation region, the maximum error is less than 6%. This implies that the analytical expression for pressure gradient works well for the values of yield stress till 2.4.
(iii) Effects of parameters such as power law index, consistency index, yield stress, stenotic height, dilatation depth, and taper angle on the above mentioned physiologically important quantities are studied.
(iv) For given value of power law index (*n*), Herschel-Bulkley fluid has greater wall shear stress than the power law fluid.
(v) It is important to note that increase in yield stress leads to increase in wall shear stress and resistance to flow.
(vi) Flow resistance increases significantly as the stenotic height increases for given *n*, *k*.
(vii) It is observed that the parameters *k* and *n* are weak parameters in the sense that these parameters bring a small change in the stream line pattern in comparison with the yield stress and the stream line pattern for tapered dilatation tube is of opposite behaviour as compared to tapered stenosed tube.
(viii) From the present work, results for power law (taking *τ*_*y*_ = 0), Bingham-plastic (taking *n* = 1), and Newtonian fluids (taking *n* = 1 and *τ*_*y*_ = 0) can be obtained.
(ix) Results illustrated through graphs show the effects of multiple diseased portions of artery in close proximity to each other (a poststenotic dilatation) on the increase of flow resistance causing the reduction of blood flow.

## Figures and Tables

**Table 1:** A comparison between numerical value of pressure gradient and approximate value of pressure gradient at the midpoint of stenotic region for different values of yield stress taking *n* = 0.8, *k* = 1.2, ζ = 0.01, and δ_1_ = 0.2.

τ_*y*_	0.05	0.1	0.4	0.8	1.2	1.6	2	2.4
Numerical value of *q*(*z*)	17.9423	18.1099	19.1145	20.4511	21.7841	23.1127	24.4368	25.7559
Analytical value of *q*(*z*)	17.9424	18.1104	19.124	20.4895	21.8712	23.2692	24.6834	26.1137

**Table 2:** A comparison between numerical value of pressure gradient and approximate value of pressure gradient at the midpoint of dilatation region for different values of yield stress taking *n* = 0.8, *k* = 1.2, ζ = 0.01, and δ_2_ = −0.2.

τ_*y*_	0.05	0.1	0.4	0.8	1.2	1.6	2	2.4
Numerical value of *q*(*z*)	5.0954	5.2106	5.9001	6.8133	7.7185	8.6152	9.5037	10.3845
Analytical value of *q*(*z*)	5.0956	5.2116	5.9163	6.8799	7.8709	8.8892	9.9349	11.008
